# A Close Association between Body Weight, Health-Related Quality of Life, and Risk Behaviors in a Sample of Italian High School Students

**DOI:** 10.3390/nu15245107

**Published:** 2023-12-14

**Authors:** Maria Francesca Lodovica Lazzeri, Francesca Mastorci, Paolo Piaggi, Cristina Doveri, Irene Marinaro, Gabriele Trivellini, Anselmo Casu, Caleb Devine, Lamia Ait-Ali, Cristina Vassalle, Alessandro Pingitore

**Affiliations:** 1 Clinical Physiology Institute, CNR, Via Moruzzi, 56124 Pisa, Italy; m.francescalodovicalazzeri@gmail.com (M.F.L.L.); cristina.doveri@cnr.it (C.D.); irene.marinaro@cnr.it (I.M.); gabriele.trivellini@cnr.it (G.T.); webmaster@easywebmaster.eu (A.C.); caleb.devine.cd@gmail.com (C.D.); lamia.ait-ali@cnr.it (L.A.-A.); alessandro.pingitore@cnr.it (A.P.); 2 Department of Information Engineering, University of Pisa, 56126 Pisa, Italy; paolo.piaggi@unipi.it; 3 Fondazione Toscana Gabriele Monasterio, 56124 Pisa, Italy; cricca@ftgm.it

**Keywords:** health, body weight, well-being, adolescents, HRQoL, school

## Abstract

Introduction: Adolescents experience rapid physical, cognitive, and psychosocial growth with different factors contributing to health and well-being. In this view, an important role is played by body weight and related perceptions. The purpose was to determine, in a sample of Italian high school students, whether health-related quality of life (HRQoL) is associated with the different weight status categories (underweight, normal weight, overweight, obese), even considering sex differences. Material and methods: Data were collected from 1826 adolescents (n = 735 males). HRQOL was analyzed using the Italian version of KIDSCREEN-52. Results: Overweight adolescents showed reductions in psychological well-being (*p* < 0.05) and self-perception (*p* < 0.05) compared with individuals in other BMI categories. Subjects with obesity reported increased bullying victimization (*p* < 0.05) and reductions in self-perception and eating disorders (*p* < 0.001), while underweight individuals were characterized by altered adherence to the Mediterranean diet (*p* < 0.001), eating disorders (*p* < 0.001), and problematic use of social media (*p* < 0.05). No sex differences were found, except for socio-economic status perceptions, where underweight girls reported higher economic well-being than boys (*p* < 0.05). Conclusions: Our findings may suggest that there is an association between weight status categories and HRQoL that is more pronounced in underweight and overweight adolescents. The association between BMI categories and psychosocial dimensions opens the need to define specific domains on which such preventive interventions should focus, always through a personalized perspective.

## 1. Introduction

Adolescence, the stage of life between childhood and adulthood, is traditionally considered a healthy stage of life; however, it represents a vulnerable period for psychological, physical, and social outcomes [1]. Adolescents experience rapid physical, cognitive, and psychosocial growth, and these neurobiological changes predispose them, on the one hand, to behavioral patterns related to diet, substance abuse, and lifestyle habits that can protect their health, but on the other hand, these same behaviors can represent risk factors and, in the medium and long-term future, can lead to disease. Generally, numerous variables that are essentially linked to lifestyle habits or the social context protect or undermine adolescents’ health. Among the factors influencing perceptions on health and well-being, an important role is played by body weight and its related perceptions, in the cases of both underweight and overweight individuals [2,3]. It is well-recognized that overweight and obesity in adolescents is associated with an increased risk of developing psychological diseases and behavioral, social, and emotional problems with consequent impairment of the quality of life [4]. Less evidence is present regarding the psychosocial effects of being underweight, although it seems to indicate the internalization of problems, in particular, depression and socially withdrawn behavior [5]. However, underweight girls usually present with greater perceptions of well-being. This may contribute to the onset of eating disorders, characterized by altered self-esteem, body image, and social media use [6,7]. The body image (BI) construct, considered in recent years to be one of the most influential factors affecting psychosocial well-being [8], is associated with body weight perceptions. This is because body image is composed of different facets: perceptual, affective, cognitive, and behavioral [9]. Moreover, an altered body image is related to unhealthy weight control behaviors, lower levels of physical activity, and reduced social relationships [10]. On the contrary, a correct body image has a protective role towards lifestyle habits and reduces the possibility of suffering from being underweight and overweight [11].

Besides lifestyle habits, which are traditionally linked to diet and physical activity, the literature has made an effort to find other moderators of body weight control, referring, for instance, to classic risk behaviors, but the evidence is still fragmentary.

Thus, taking into account these aspects, the present study, conducted on a sample of Italian high school students, has the purpose of determining whether there are health-related quality of life (HRQoL) changes according to the different body weight categories (underweight, normal weight, overweight, obese), even considering sex differences. We hypothesized, in fact, that underweight, overweight, and obese individuals would show reductions in HRQoL compared to their normal weight counterparts. Second, we considered the possible sex differences involved in this relationship.

## 2. Materials and Methods

### 2.1. Partecipants and Procedures

Data were collected between 2022 and 2023 from the platform of the AVATAR project “A new purpose for promotion and eVAluation of healTh and well-being Among healthy teenageRs” developed by the Institute of Clinical Physiology of the CNR, involving a sample of 1908 students [12]. Of the initial population, 82 students were excluded for the following reasons: diagnosed neuropsychiatric or other diseases (n = 9), the absence of signed informed consent (n = 48), questionnaires not filled in (n = 15), and Internet connection problems (n = 10). Therefore, the final population consisted of 1826 adolescents. Participants were instructed on how to complete the questionnaires, and all tests were performed during school hours. In every school class, all the adolescents filled out the questionnaire, and when they were not eligible due to exclusion criteria reasons, they were excluded from the study retrospectively. Participants were previously instructed on how to complete the questionnaires and conduct the tests. One or two project members visited each school to provide the adolescents with verbal and written information about the data collection. All tests were conducted during participants’ computer lessons during school time. No incentive was provided to adolescents or parents. A research assistant was available to provide information and technical support to help the students to complete the questionnaires.

### 2.2. Ethics

All procedures performed in the study were conducted in accordance with the ethical standards of the institutional and/or national research committee and with the 1964 Helsinki Declaration and its later amendments or comparable ethical standards. The AVATAR project has been accepted by the Regional Pediatric Ethics Committee (Azienda Ospedaliero Universitaria Meyer) (code 166/2018).

### 2.3. Measures

Weight and height data were collected through self-assessments. The body mass index (BMI) was calculated as weight (kg)/height (m^2^). Adolescents aged <19 years were classified into four weight categories, underweight, normal weight, risk of overweight, and obesity, based on the BMI cut-off points for children, approved by the International Obesity Task Force (IOTF) [13]. Adolescents aged >19 years were instead classified into four weight categories according to the traditional nomenclature, underweight, normal weight, overweight, and obesity based on the adult BMI cut-off points [14]. Data concerning risk factors were collected through self-assessments through a series of questions. In particular, questions were administered with dichotomous “yes/no” answers concerning smoking, cannabis use, substance use, psychotropic drug use, alcohol consumption, sexual intercourse, and contraceptive use. If the response to the presence of one of these risk behaviors was positive, the frequency with which this behavior was practiced was also asked about.

The Italian version of KIDSCREEN-52 was used to assess the health-related quality of life (HRQoL) [15,16]. KIDSCREEN is a self-report questionnaire designed to assess health-related quality of life. It aims to monitor and measure the personal experiences of children and adolescents about their perceptions of their health status and well-being. The questionnaire, which describes physical, psychological, mental, social, and functional aspects of well-being, consists of 52 items grouped in 10 dimensions (physical well-being, psychological well-being, moods and emotions, self-perception, autonomy, parent relations, and home life, social support and peers, school environment, social acceptance (bullying), financial resources) [15,16]. Some sample items are “In general, how would you say your health is?” for the physical well-being dimension; “Have you felt satisfied with your life?” for moods and emotions; and “Have you been happy with the way you are?” for self-perception. Cronbach’s alpha scores range from 0.77 to 0.89 for the dimensions of the 52-item version. Excluding the mood and bullying dimensions, higher values of the variables express a better health-related quality of life. KIDSCREEN questionnaires are psychometrically tested using data from a multicenter European study that included a sample of 22,827 children recruited from 13 countries [17].

In more detail, physical well-being explores the levels of an adolescent’s physical activity, energy, and fitness; psychological well-being examines the psychological well-being of the adolescent, including positive emotions and satisfaction with life; mood and emotions cover how much the adolescent experiences depressive moods and emotions and stressful feelings; self-perception includes whether the appearance of the body is viewed positively or negatively; autonomy looks at the opportunity given to an adolescent to create his/her social and leisure time; parent relations examine the relationship between the parents and the atmosphere in the adolescent’s home, with a focus on the quality of the interactions between the adolescent and parent or carer; social support and peers considers the nature of relations with friends and peers; school environment describes an adolescent’s perceptions of their cognitive capacity, learning, and concentration; social acceptance reflects the feeling of being rejected by peers in school; and financial resources describes the quality of the perceived financial resources [15,16].

Dietary practices were evaluated by means of the Mediterranean Diet Quality Index for children and adolescents (KIDMED) [18]. The KIDMED index is based on principles related to Mediterranean dietary patterns as well as those that undermine them (for example, “Every day I eat fruit or freshly squeezed fruit juice”, “Regularly once a day would consume fresh and cooked vegetables”, “I eat pasta and rice almost every day (5 or more per week)”. The index ranges from 0 to 12 and consists of a self-administered 16-question test. The validity of the KIDMED index is demonstrated by evidence that a higher score is associated with the expected patterns of food and nutrient intake that are representative of a good quality diet (Cronbach’s alpha = 0.79, 95% CI: 0.71–0.77). Scores of 0 to 3 reflect a poor diet, while values ranging from 4 to 7 and 8 to 12, respectively, describe medium and high adherence. Physical activity levels were assessed using the Physical Activity Questionnaire for Older Children (PAQ-C). The questionnaire provides a general amount of physical activity for 8- to 20-year-olds. The PAQ-C is a self-administrated questionnaire consisting of nine items rated on a five-point scale (e.g., “In the last 7 days, during your physical education (PE) classes, how often were you very active (playing hard, running, jumping, throwing)?” “In the last 7 days, on how many evenings did you do sports, dance, or play games in which you were very active?” “On the last weekend, how many times did you do sports, dance, or play games in which you were very active?”). The average score of the items is used to create the final PAQ summary score, where higher scores indicate more active children/adolescents. Previous studies support the validity of the PAQ instrument for assessing general levels of physical activity. Validation studies have found the PAQ-C to be highly reliable (Cronbach alpha scores ranged from 0.72 to 0.88) [19].

Involvement in the individual’s dependence on various social media platforms, such as Facebook, Twitter, Instagram, Snapchat, and YouTube, over the past year were evaluated with the Bergen Social Media Addiction Scale (BSMAS). The BSMAS contains six items reflecting core addiction elements (salience, mood modification, tolerance, withdrawal, conflict, and relapse) (i.e., “You spend a lot of time thinking about social media or planning how to use it”, “You feel an urge to use social media more and more”) [20]. Total possible scores range from 6 to 30, with higher scores indicating more problematic social media use. Regarding the threshold between non-disorder and disorder, recently, Luo and colleagues proposed a score of 24 as a clinical cut-off point, based on the gold standard for clinical diagnosis [21].

Eating attitudes and behaviors were assessed using EAT-26, which is used in many studies as a screening tool to identify people with eating disorders at an early stage. It consists of three subscales: dieting, eating preoccupation and oral control (i.e., “I am terrified about being overweight”, “I avoid eating when I am hungry”, “I find myself preoccupied with food”) [22]. The total score (between 0 and 78) provides an overall risk score, where higher scores indicate a greater risk of an eating disorder. Total scores of 20 or above are considered to be in the clinical range.

### 2.4. Statistical Analysis

The statistical data analysis was performed using SPSS 27 software. Data are presented as the mean ± SD. Categorical variables were presented as counts and percentages. A *p*-value ≤ 0.05 was considered statistically significant. The Shapiro–Wilk test was used to assess the normality of the data distribution for continuous variables before parametric analyses. Differences in BMI and HRQoL by sex were analyzed using Student’s unpaired *t*-test. A two-way analysis of variance (ANOVA) was used to examine differences in the KIDSCREEN-52 dimension scores across sex and BMI categories (assuming a linear trend), while also testing for sex*BMI interaction effects. Levene’s test was used to assess the homogeneity of variances across gender groups and BMI categories, and the Welch test was used in the presence of heteroscedasticity. Sensitivity analyses performed after combining overweight subjects with subjects with obesity yielded similar results. A *p*-value ≤ 0.05 was considered statistically significant.

## 3. Results

### 3.1. Socio-Demographic Features of the Study Sample

The final population consisted of 1826 adolescents (from 14 to 20 years old). Table 1 displays the demographic characteristics and BMI categories of the entire cohort of 1826 participants (60% girls) included in the analyses. On average, boys had higher BMIs compared to girls (*p* < 0.01).

When we considered the BMI categories, expressed as the percentages of boys and girls who were underweight, normal weight, overweight, or obese, boys were more likely to be overweight or obese, while girls were more likely to be underweight (*p* < 0.001).

### 3.2. Sex Differences on HRQoL and Risk Behaviors

Descriptive results of the health-related quality of life in our population divided by sex are shown in Table 2. Regarding HRQoL, several variables differed significantly according to sex. Compared to girls, boys perceived themselves to have better physical well-being (*p* < 0.001), emotional status (*p* < 0.001), self-perception (*p* < 0.001), autonomy (*p* < 0.001), relationships with parents (*p* < 0.001), and school environments (*p* = 0.01). Regarding risk behaviors (Table 3), boys reported more pronounced adherence to the Mediterranean diet than girls (*p* < 0.001) and normal use of social networks, while their female counterparts showed problematic use (*p* < 0.001). There were also no perceptions of eating disorders, in contrast to girls (*p* < 0.001).

### 3.3. Relationship between Weight Status Categories and HRQoL in the Total Population and by Sex

The results of the analysis of health-related quality of life in the total population and by sex according to BMI categories are shown in Table 4. In our sample, an individual’s BMI category influences their perception of psychological well-being (*p* = 0.04). In particular, overweight subjects showed lower scores in this dimension compared to their normal weight counterparts. BMI categories are also associated with self-perception (*p* = 0.003), especially overweight and obese adolescents reported lower scores compared to underweight and normal weight subjects. Also, BMI was inversely associated with the perceived quality of financial resources (*p* < 0.001), with underweight and normal weight individuals having higher scores compared to overweight and obese individuals. BMI classification was correlated with peer relationships (*p* < 0.001) and bullying behavior (*p* = 0.003); in both categories, underweight and obese adolescents reported lower levels of these variables. For lifestyle habits, physical activity (*p* = 0.002) and adherence to the Mediterranean diet (*p* = 0.001) correlated with BMI classes; underweight students showed reductions in these healthy behaviors as compared to individuals in the other categories. Examining the impacts of gender and BMI categories on HRQoL levels, only the perception of financial resources decreased with an increasing BMI, but this decrease was more pronounced in females than in males (*p* = 0.001).

### 3.4. Relationship between Weight Status Categories and Risk Behaviors in the Total Population and by Sex

Data on the analysis of risk behaviors in the total population and by sex as a function of BMI categories are shown in Table 5. In our population, an individual’s BMI category influences both their adherence to the Mediterranean diet (*p* = 0.001) and physical activity (*p* = 0.002). In particular, both underweight individuals and adolescents with obesity showed reduced adherence compared to the other categories. Also, BMI categories are associated with social addiction (*p* < 0.05), revealing that normal weight adolescents had more altered behaviors, showing more problematic use of social network than those from other categories. Moreover, BMI is linked with perceptions on eating disorders (*p* < 0.05), especially in underweight subjects and in subjects with obesity. When we assessed the impacts of sex and BMI categories on risk behaviors, none of the possible risk factors (diet, exercise, social addiction, eating disorders) showed significant differences.

## 4. Discussion

This research aimed to determine the effects of belonging to a specific BMI category on the health-related quality of life (HRQoL) in a sample of late adolescent students, taking sex differences into account. The main results of this study can be summarized by the following points: (i) males are more likely be in the overweight and obesity category, while girls are more highly represented in the underweight category; (ii) boys showed higher values of perceived well-being, better emotional states and self-perception, and better family and school relationships, while females reported problematic use of social networks and a greater tendency for eating disorders.

When we consider BMI categories, (i) reduced perceptions of psychological well-being, self-perception, and fewer social relationships were found in the overweight and obese adolescent categories, while the underweight category was characterized by better adherence to the Mediterranean diet and better perceptions of financial resources, especially in girls; (ii) normal weight subjects exhibited problematic use of social networks, whereas eating disorders were more pronounced in underweight and obese adolescents.

Consistent with the literature, the present results provide clear evidence that overweight and obese individuals are more common in the male population. This prevalence is usually stronger in high income and upper middle income countries, where the prevalence of obesity is two-fold greater in boys than in girls [23]. This sex difference may be due to biological influences. In fact, biological differences in body composition between sexes, which are already present during childhood, became more marked in adolescence by the role of sex hormones [24,25]. In fact, females have higher levels of circulating concentrations of leptin, a hormone that is responsible for increased appetite suppression and the promotion of energy utilization [26]. Another possible explanation for the sex differences in obesity seems to be related to brown adipose tissue (BAT), whose decrease is implicated in the development of obesity [27]. Although there is no clear evidence, some studies have found BAT to be more prevalent in girls than in boys [28]. Furthermore, it is important to emphasize that the number of school-aged adolescents with obesity is expected to rise from 150 million worldwide to over 250 million by 2020, with a long-term increase in other related chronic disease, such as type 2 diabetes and cardiovascular disease [23,29]. This growing prevalence of obesity in adolescence globally is a major challenge, not only due to the long-term effects, but also due to the short-term health complications, including increased cardiovascular risk, disturbances of sex hormones, and hepatic and orthopedic problems [30]. Although the physiological underlying mechanisms responsible for the rise in adolescent obesity are not yet fully understood, certainly, adolescence is a time of rapid physical, social, and psychological development, and as a result, it offers multiple possibilities for unhealthy and/or risk behaviors. In this frame, psychological/mental health problems that emerge during this period can often contribute to physical health outputs and persist into adulthood, creating a vulnerability substrate or altering adolescents’ well-being and quality of life [31,32]. Independently of body weight, our results show an impairment in HRQoL dimensions, in line with the view that adolescence is not necessarily the age of highest health, but rather a period involving the onset of risk factors; this deterioration is more pronounced in girls than in boys [6,33,34].

To try to explain this vulnerability, also in line with previous literature and our aforementioned results, several theories have been formulated [35,36]. Probably, the different psychosocial pictures between sexes can be explained by the time of pubertal changes in boys and girls, in which girls enter the state of physiological alteration before boys with associated physical and hormonal variations [37]. This female development is associated with an effect on femininity and thus with psychological outputs such as depressive behaviors, which are also linked to the time of hormonal maturation.

Within this susceptible framework, it is necessary to highlight that adolescents are more prone to engagement in risky behaviors, including abnormal perceptions of one’s body weight and thus, an alteration of one’s image. In this field, our previous data collected in a sample of early adolescents demonstrated that weight status correlated more with the psychological dimension in girls, whereas in boys, a stronger association between weight and physical status was observed [6].

The possible association between body weight control and the psychosocial profile creates a vicious circle in which weight stigma may reinforce a poor body image, resulting in an increase in stress levels associated with reduced emotion regulation, less rational decision-making, and engagement in higher risk behaviors [38,39]. It is not a coincidence that, during adolescence, everything to do with the body becomes relevant, with important implications on mental health-related aspects that are reinforced by the internalization of the esthetic model imposed by society through social media [40]. Not surprisingly, the increased stigmatization of overweight/obesity in the last few years may be related to more psychological distress and risky behaviors, as evidenced by our results, when we analyzed health-related quality of life dimensions according to BMI categories. In fact, compared to their normal weight peers, adolescents who are overweight/obese had reduced perceptions of their psychological well-being, low self-esteem, reduced social relationships with peers, and greater perceptions of being bullied. In line with our results, these alterations may be amplified in the case of self-perceptions of being overweight, considered to be a predictor of maladaptive coping following stressful events [41]. In fact, evidence demonstrates that self-perception of being overweight is more powerfully linked with a reduced health-related quality of life and engagement in risky behaviors [41]. Usually, it is well-known that overweight or obese adolescents have an impairment in their quality of life, characterized by elevated symptoms of depression, emotional difficulties, lower self-esteem, and higher school dropout rates [42,43]. However, little is known about the other side of the coin, i.e., underweight adolescents, although this affects 5% to almost 12% of adolescents, apart from disordered eating, body dissatisfaction, and altered social relationships, as also highlighted in our results [44]. One of the factors linked to body weight, mainly in the underweight category, is body image and thus body dissatisfaction, which is considered to be one of the most influential factors affecting well-being in adolescents [45]. Body dissatisfaction is prevalent among adolescents, and recent data show that 24% of normal weight girls and 22% of normal weight boys were dissatisfied with their bodies, while in overweight girls and boys, low-level body satisfaction reached 59% and 48%, respectively [46]. The data presented here, which clearly show impairments in well-being and quality of life, especially in individuals in the underweight and obese categories, are, however, perception data and can certainly impair body image. It is not a coincidence that the ‘self-perception’ dimension is lower in overweight and obese individuals. Although no sex differences in body-image-related dimensions were revealed in our data, usually, underweight girls have been shown to have higher rates of body satisfaction as compared to their normal and overweight counterparts [46]. While previous data demonstrated a sex effect in this vicious circle, our underweight female population reported higher perceived economic well-being as compared with boys. There is no clear evidence of this association, since few studies have studied the relationship of these dimensions with socio-economic status (SES) or perceptions of socio-economic status. The few data present are conflicting, showing, for example, that unhealthy weight control is not limited to upper socio-economic groups, and in other cases, dietary behavior and attempts to lose weight are more typical of girls of higher SES [47,48]. Certainly, social and economic inequalities cause food and body weight inequalities [49]. In this respect, there is evidence that girls from families of high socio-economic status estimated their weight more correctly than female students of middle and low status, pointing to the ‘mother’s education’ factor as being responsible for this association [50].

Not surprisingly, our results suggest that underweight adolescents, regardless of gender, have problematic use of social networks. Social media platforms, often accompanied by interactive modes such as likes, comments, and stories, are associated with emotional ups and downs with satisfaction and/or inadequacy, particularly in girls [51]. Furthermore, data obtained from principal social media platforms show that altered social media use may induce poor body image and self-perception characteristics and eating disorders, as suggested also by our results [52]. In addition, screen time on social media has been related to an unrealistic view of beauty standards, leading to subsequent eating disorders and depressive behaviors [53].

This study has some strengths and limitations. The strength of this study is that little is known about the relationship between HRQoL and body weight categories in Italian late adolescents, despite the increase in Italy and other industrialized countries of overweight or obesity, probably due to a decrease in adherence to the Mediterranean nutritional lifestyle [54]. More precise knowledge of this relationship would allow the implementation of targeted and customized preventive strategies based on both the psychosocial profile of the adolescent and considering a parameter as simple as the BMI, which is considered to be a potential predictor of health-related quality of life in adolescents, as reported in our previous study [6].

One of the limitations of the study may be represented by the calculation methodology used for the BMI because it does not discriminate between lean and fat mass and does not classify the type of obesity. In addition, the use of self-reports for psychological evaluations could also be a limitation. Moreover, perceptions of well-being and health status may vary from day to day, especially in adolescence, when they are related to the different emotional backgrounds; thus, the results must not be considered in an absolute way, but limited to a specific time window. Finally, the different numbers of subjects in the different BMI categories, in particular, the reduced number of adolescents with obesity, may not show certain significant differences that would be visible in a larger sample.

## 5. Conclusions

In conclusion, this study proposes that there is a relationship between body weight expressed in categories and some dimensions of HRQoL and that this association is more pronounced in overweight and obese adolescents, irrespective of sex. Also, it is interesting that BMI categories, particularly overweight and obese, correlate with reductions in psychological well-being, self-perception, and bullying. Another very important result, in our opinion, is that underweight adolescents showed higher economic well-being and greater prevalence of eating disorders, and problematic use of social media. Our data suggest the importance of and the need to implement weight control strategies that take into account not only metabolic parameters but also psychological dimensions. These factors should be taken into consideration when designing programs for adolescents with weight-related problems, although the variety of psychological problems associated with being overweight or underweight is so wide that there is a risk of the intervention not being too specific and focused on the real problem. This opens up the need for the scientific community to define, if possible, specific domains in which such interventions should focus and which may be the most beneficial, always through a personalized perspective [55].

## Figures and Tables

**Table 1:** Demographic characteristics and BMI classification of the study population (total and by sex).

Variables	Total (n = 1826)	Boys (n = 735)	Girls (n = 1091)	*p* Value
Age, (years)	16.85 ± 1.49	16.92 ± 1.52	16.81 ± 1.46	ns
BMI (kg/m^2^)	21.33 ± 4.64	21.69 ± 3.23	20.71 ± 2.80	<0.01
Underweight	120 (6)	51 (7)	69 (6)	
Normal weight	1511 (83)	576 (78)	935 (86)	
Overweight	148 (8)	76 (10)	72 (7)	
Obesity	47 (3)	32 (4)	15 (1)	<0.001

Data are expressed as the mean ± standard deviation (SD) or number (%). BMI: body mass index, ns: not significant. For BMI categories, *p* refers to the χ^2^ test.

**Table 2:** Sex differences in health-related quality of life variables.

Variables	Boys (n = 735)	Girls (n = 1091)	*p*-Value
Physical well-being	57.93 ± 17.01	44.23 ± 16.09	<0.001
Psychological well-being	36.45 ± 6.5	34.05 ± 6.93	<0.001
Mood/Emotion	43.2 ± 8.98	38.34 ± 9.05	<0.001
Self-perception	45.68 ± 7.84	40.14 ± 8.79	<0.001
Autonomy	42.34 ± 9.76	38.99 ± 8.99	<0.001
Parent relationship	44.55 ± 10.09	42.07 ± 10.66	<0.001
Financial resources	48.56 ± 9.71	48.79 ± 9.61	0.634
Peers	45.88 ± 10.49	45.18 ± 9.81	0.156
School environment	42.31 ± 7.79	41.39 ± 7.03	=0.01
Social acceptance (bullying)	49.53 ± 10.87	49.79 ± 9.9	0.608

Data are given as the mean ± SD (95% CI). Data for the KIDSCREEN-52 dimension are calculated as the mean T-scores according to the KIDSCREEN group. *p*-values were calculated via the Student’s unpaired *t*-test.

**Table 3:** Sex differences in risk behaviors.

Variables	Boys (n = 735)	Girls (n = 1091)	*p*-Value
KIDMED	8.06 ± 3.33	7.87 ± 2.62	<0.001
PAQ-C	2.55 ± 0.72	2.14 ± 0.69	0.329
BSMAS	12.95 ± 4.51	15.03 ± 5.22	<0.001
EAT-26	10.62 ± 11.11	13.07 ± 12.13	<0.001

Data are given as the mean ± SD (95% CI). KIDMED: Mediterranean Diet Quality Index for children and adolescents; PAQ-C: Physical Activity Questionnaire for Older Children; BSMAS: Bergen Social Media Addiction Scale; EAT-26: Eating attitudes and behaviors. *p*-values were calculated via the Student’s unpaired *t*-test.

**Table 4:** BMI categories according to health-related quality of life dimensions in the total population and by sex.

Total Population	Physical Wellbeing	Psychological Wellbeing	Mood Emotion	Self-Perception	Autonomy	Parent Relationship	Financial Resources	Peers	School Environment	Bullysm
Underweight 120 (6)	46.1 ± 18.1	34.2 ± 8.3	39.9 ± 11.2	42.2 ± 10	39.1 ± 10.9	42.5 ± 12.2	49.1 ± 10.8	42.8 ± 9.4	41.7 ± 8.7	48.1 ± 10.8
Normal weight 1511 (83)	50.2 ± 26.9	35.2 ± 6.5	40.3 ± 9.1	42.6 ± 8.7	40.5 ± 9.1	43.3 ± 10.4	49 ± 9.5	46 ± 9.9	41.8 ± 7.2	50 ± 10.1
Overweight 148 (8)	47.4 ± 26.5	33.8 ± 8.5	39.7 ± 10.2	40.7 ± 9.6	39.3 ± 9.7	41.9 ± 10.2	45.9 ± 9.4	43.3 ± 11.3	41.3 ± 7.5	49.3 ± 10.9
Obesity 47 (3)	49.1 ± 36.4	34 ± 7.9	40.8 ± 8.5	38.8 ± 6.1	39.8 ± 13.9	41.3 ± 11.4	46.1 ± 9.2	42.9 ± 11.7	41.7 ± 7.8	45.0 ± 11.8
*p*	0.30	=0.04	0.82	=0.003	0.20	0.29	<0.001	<0.001	0.89	=0.003
BOYS										
Underweight 52 (8)	51.5 ± 24.8	36.9 ± 7.7	43.5 ± 11.5	45.8 ± 7.3	40.5 ± 12.3	43.4 ± 11.9	47.4 ± 12	42.7 ± 9.2	42.1 ± 9.9	47.9 ± 10.5
Normal weight 577 (78)	44.6 ± 16	36.6 ± 6.1	43.4 ± 8.8	46 ± 7.9	42.7 ± 9.3	45 ± 9.9	49 ± 9.5	46.7 ± 10.4	42.2 ± 7.6	49.8 ± 10.9
Overweight 73 (10)	51.9 ± 29.5	35.3 ± 7.8	42.3 ± 8.5	45.2 ± 8.2	41.3 ± 9.8	40.8 ± 11.1	46.5 ± 9.6	43 ± 10.1	42.9 ± 7.3	49.8 ± 10.9
Obesity 33 (4)	36.5 ± 8.1	35.1 ± 8.6	42.1 ± 9.2	40.8 ± 5.4	41.8 ± 13	42.9 ± 12.8	46.9 ± 9.2	42.3 ± 12.3	42.3 ± 8.4	46 ± 10.9
GIRLS										
Underweight 69 (6)	42.2 ± 9	32.3 ± 8.3	37.3 ± 10.3	39.6 ± 10.9	38 ± 9.7	41.9 ± 12.4	50.3 ± 9.8	42.9 ± 9.7	41.3 ± 7.7	48.2 ± 11.1
Normal weight 935 (86)	59.4 ± 36.9	34.4 ± 6.6	38.5 ± 8.8	40.6 ± 8.6	39.2 ± 8.8	42.2 ± 10.5	49 ± 9.6	45.5 ± 9.6	41.5 ± 7	50.1 ± 9.6
Overweight 72 (7)	42.7 ± 22.1	32.3 ± 9	37 ± 11.1	36 ± 8.7	37.2 ± 9.3	42.9 ± 9.1	45.2 ± 9.1	43.5 ± 12.4	39.7 ± 7.4	48.7 ± 11
Obesity 15 (1)	55.4 ± 43	31.7 ± 5.8	38.1 ± 6.2	34.7 ± 5.6	36 ± 15.2	38.1 ± 7.1	44.5 ± 9.4	44 ± 10.8	40.7 ± 6.4	42.9 ± 13.8
*p* (BMI trend) adj. for sex	0.25	0.48	0.66	0.33	0.31	0.08	*p* = 0.001	0.50	0.68	0.08

Data are given as the mean ± SD or number (%). Data for the KIDSCREEN-52 dimension were calculated as the mean T-scores according to the KIDSCREEN Group. In the total population, *p* is representative of the post-hoc test, for sex-related differences, *p* is representative of the BMI trend adjusted for sex.

**Table 5:** BMI categories according to risk behaviors in the total population and by sex.

Total Population	KIDMED	PAQ-C	BMAS	EAT-26
Underweight 115 (7)	7.2 ± 3.2	2.1 ± 0.7	14.4 ± 5.1	14.9 ± 15.7
Normal weight 1442 (83)	8.1 ± 2.9	2.3 ± 0.7	13.2 ± 5.1	11.5 ± 11.3
Overweight 140 (8)	7.5 ± 3.1	2.3 ± 0.7	13.5 ± 4.7	14.5 ± 12.3
Obesity 45 (2)	7.2 ± 3.3	2.2 ± 0.8	12.6 ± 4.6	15.4 ± 11
*p*	=0.001	=0.002	<0.05	<0.001
BOYS				
Underweight 52 (8)	6.8 ± 0.4	2.3 ± 0.1	11.5 ± 0.7	13.6 ± 1.7
Normal weight 577 (78)	8.3 ± 0.1	2.6 ± 0.1	13.3 ± 0.2	10 ± 0.5
Overweight 73 (10)	7.5 ± 0.3	2.5 ± 0.1	11.7 ± 0.6	11.8 ± 1.4
Obesity 33 (4)	7 ± 0.5	2.3 ± 0.1	11.7 ± 1	13.1 ± 2.2
GIRLS				
Underweight 69 (6)	7.4 ± 0.4	2 ± 0.1	14.4 ± 0.6	15.9 ± 1.5
Normal weight 935 (86)	7.9 ± 0.1	2.2 ± 0.1	15.1 ± 0.2	12.4 ± 0.4
Overweight 72 (7)	7.6 ± 0.4	2 ± 0.1	15.2 ± 0.6	17.3 ± 1.4
Obesity 15 (1)	7.6 ± 0.8	1.8 ± 0.2	14.1 ± 1.3	19.5 ± 3
*p* (BMI trend) adj. for sex	0.48	0.91	0.29	0.25

Data are given as the mean ± SD (95% CI). KIDMED: Mediterranean Diet Quality Index for children and adolescents; PAQ-C: Physical Activity Questionnaire for Older Children; BSMAS: Bergen Social Media Addiction Scale; EAT-26: Eating attitudes and behaviors. In total population *p* is representative of the post-hoc test; for sex-related results, *p* is representative of the BMI trend adjusted for sex.

## Data Availability

The datasets used and/or analyzed during the current study are available from the corresponding author on reasonable request.
